# Construct validity of the German version of the Emotion Reactivity Scale

**DOI:** 10.1186/s40359-023-01458-y

**Published:** 2023-12-02

**Authors:** Antonia M. Lüönd, Martina F. Thoma, Tobias R. Spiller, Sonja Weilenmann, Billy Jansson, Monique C. Pfaltz

**Affiliations:** 1 Medical Faculty, University of Zurich, Zurich, Switzerland; 2 Department of Consultation-Liaison Psychiatry and Psychosomatic Medicine, University Hospital Zurich, Zurich, Switzerland; 3 Department of Psychology and Social Work, Mid Sweden University, Kunskapens Väg 1, Östersund, Sweden

**Keywords:** Emotion reactivity scale, Emotion regulation, Emotion sensitivity, Emotion intensity, Emotion persistence

## Abstract

**Background:**

Emotional reactivity is an important construct to consider when studying mental disorders. This study was conducted to translate and assess the factor structure, construct validity and internal consistency of a German version of the Emotion Reactivity Scale (ERS), which is an originally English questionnaire assessing three components of emotional reactivity: sensitivity, intensity and persistence of emotions.

**Methods:**

The German ERS and a range of questionnaires used to assess convergent and discriminant validity were completed by 334 German speaking Swiss participants.

**Results:**

Confirmatory factor analysis showed strong support for a bi-factor model, with evaluation indices pointing to a unidimensional construct rather than to domain specific factors. The questionnaire showed good reliability and the factor structure was similar across gender. The ERS showed convergent validity with general psychopathology, behavioral inhibition, negative affect, orienting sensitivity, depressive symptoms and symptoms of disordered eating, and discriminant validity with behavioral activation and alcohol consumption.

**Conclusions:**

Findings support the construct validity of the German ERS and suggest that it assesses a unidimensional construct with high internal consistency. Accounting for the unidimensional nature of the scale and aiming for efficient assessment tools, future research could, based on these findings, develop and evaluate the psychometric properties of a short version of the ERS.

**Supplementary Information:**

The online version contains supplementary material available at 10.1186/s40359-023-01458-y.

## Introduction

Emotional reactivity is defined as the extent to which an individual experiences emotions (a) in response to a wide array of stimuli (i.e., emotion sensitivity), (b) strongly or intensely (i.e., emotion intensity), and (c) for a prolonged period of time before returning to baseline level of arousal (i.e., emotion persistence) [1]*.* Nock and colleagues [1] proposed that difficulties in emotional reactivity might predispose individuals to having emotion regulation difficulties, which are a transdiagnostic characteristic of many psychiatric disorders [2–4] and important for the wellbeing of those affected by psychopathology [5]. Consequently, emotional reactivity is an important construct to consider when studying emotion regulation and psychopathology. Notably, levels of emotional reactivity can differ across psychopathologies. For example, while anxiety disorders have been linked to emotional hyperreactivity, antisocial personality disorder is associated with emotional hyporeactivity [6]. Furthermore, emotional reactivity mediates the relationship between several psychopathologies and self-injurious thoughts and behaviors [1]. Non-suicidal self-injury is a highly prevalent and distressing experience [7]. It has been associated with suicidal attempts [8, 9] and contributes to physical harm that may require medical intervention. Given the potential for emotional reactivity to change over time [10], recognizing individuals with heightened emotional reactivity early in clinical practice could help prevent the development and maintenance of dysfunctional coping strategies like non-suicidal self-injury [11].

Emotional reactivity can be measured using the Emotion Reactivity Scale (ERS) developed by Nock and colleagues [1]. The ERS can be used in in both research and clinical settings and comprises 21 questions categorized into three subscales: emotion intensity (EI) emotion sensitivity (ES) and emotion persistence (EP). The internal consistency of the English version, both for the total ERS score and its subscales, is good (Cronbach’s α = 0.94 for total ERS score, α = 0.88 for Sensitivity, α = 0.86 for Intensity, α = 0.81 for Persistence; [1]). The ERS has so far been translated into Dutch, French and Persian. All three translated versions have shown an internal consistency comparable to the original scale [12–14]. Regarding the factor structure of the ERS, Nock and colleagues proposed a single-factor model [1], whereas Claes and colleagues as well as Izadi-Mazidi and colleagues found support for both one and three-factor structures in the Dutch and the Persian version of the ERS [12, 14]. In contrast, Lannoy and colleagues found that the French version was best described by a hierarchical model, comprising a single second-order factor with three subscales loading on a higher-order emotional reactivity factor [13]. However, in line with Nock and colleagues [1], the authors of the Dutch, Persian and French versions argue that a one-factor structure probably best characterizes the ERS [12–14]. All validation studies furthermore found good construct validity. For example, they found that higher ERS scores were associated with higher negative affect [12], behavioral inhibition, depressive symptoms and proneness to eating disorders [1, 14]. Furthermore, they found that higher ERS scores were negatively correlated with attention and behavioral control [1, 12]. Individuals with higher ERS scores might thus be more prone to negative emotional experiences, greater behavioral inhibition (such as a tendency to avoid certain situations [15]), depressive symptoms, and eating disorders, while at the same time experiencing challenges in maintaining attention and behavioral control, highlighting the role of emotion reactivity in different aspects of mental health and behavior. Notably, the ERS was only associated with some of the psychopathological symptoms measured in those studies (e.g., there was a weak association between ERS scores and substance use disorder, see [1]. This suggests that emotion reactivity may not generally be elevated in psychopathology and thus could be an important characteristic to consider in clinical research.

The objective of the current study was twofold: First, we aimed to translate the English original version [1] into German to make the scale available for assessment in German-speaking countries. Second, we sought to assess the factor structure of the German ERS through confirmatory factor analysis. Unlike previous validation studies [1, 12–14], which compared unidimensional, and three-factor correlated models, we also aimed to test a bifactor model. A bifactor model includes a general factor that loads directly onto all indicators, alongside three specific (uncorrelated) factors that load onto a subset of the same indicators [16]. Bifactor models retain a general factor but also recognize the multidimensionality. As a bifactor model includes a common factor across all indicators, it can therefore simultaneously account for unique variance within each indicator, allowing for a comprehensive assessment of the degree to which each measure assesses common versus separable constructs. Applying a bifactor approach can inform researchers on the psychometric structure of a measure, including the properties of total- and subscale scores (and whether total and/or subscale scores should be computed).

Furthermore, we aimed to evaluate the construct validity and internal consistency of the German ERS. Consistent with findings from the English, French, Persian and Dutch versions of the ERS [1, 12–14] we predicted that ERS scores would be linked to related constructs such as behavioral inhibition, symptoms of eating disorders, general psychopathology, orienting sensitivity and depressive symptoms (convergent validity) but not to unrelated constructs such as extraversion, effortful control, behavioral activation and alcohol use disorders (discriminant validity). Based on previous validation studies [1, 12–14], we expected an excellent Cronbach’s alpha (i.e., α ≥ 0.9) for both the total score and all subscales.

## Method

### Participants

We recruited a convenience sample using online platforms, mouth-to-mouth propaganda, social media, personal network of the study team and a study pool of the Department of Consultation-Liaison Psychiatry and Psychosomatic Medicine of the University Hospital Zurich. Our power analysis, conducted with the semTools R package (Version 0.5–6; [17]), determined that, given a df (186) for the three-factor model, a sample size of 143 participants was required to closely fit the model and detect model misspecifications, while 169 participants were needed for a less close fit. Sample size calculations were based on 95% power, and with an acceptable fit defined by a cut-off value of RMSEA ≤ 0.08.

Data was acquired in two waves (December 2019 – January 2020 and during August 2020) including data from all participants who fulfilled inclusion criteria. Participants had to be aged 18 to 65 and proficient in German to participate. We excluded data from 13 participants who completed the survey within less than 15 min and 11 participants who took more than 10 h. As a result, our final sample comprised 334 participants. Demographic information of all included participants is illustrated in Table 1. The study did not fall within the scope of the Human Research Act, as confirmed by the cantonal ethics committee of Zurich prior to the conduction of the study (Reference Nr. 2019–02093). All participants provided consent online prior to participating.

**Table 1:** Demographic characteristics

	M ± SD
Age (in years)	27 ± 8
Gender (*n*)	
Females	179 (53.6%)
Males	149 (44.6%)
Non-binary	3 (0.9%)
Undefined	3 (0.9%)
Education (*n*)	
Compulsory school incl. apprenticeship	39 (11.7%)
Baccalaureate / maturity/ federal diploma	142 (42.5%)
University degree (any)	153 (45.8%)

### Procedure

After obtaining approval from the original authors of the ERS, the questionnaire was translated into German. This process involved three independent native German speakers from our study team, each holding an M. Sc in Psychology. Among the translators, one was a licensed Germanic linguist, while the other two had resided in English-speaking countries for at least one year. The three translations were then compared, and discrepancies were discussed and resolved. The final German version was then back-translated by three independent native English speakers without psychological background. After discussing discrepancies between translated versions and possible deviations from the original questionnaire, a final German questionnaire was created.

Data were collected online, and the survey was programmed using *Remark Web Survey Version 5*. After accessing the survey, participants were informed about the content of the study and the inclusion criteria. Informed consent was obtained from all participants on the first screen. Completion of the survey took about 30 – 45 min. After completion of all questionnaires, participants received a compensation of 20 Swiss francs. 

### Measures

The final version of the translated German version of the Emotion reactivity scale (ERS; [1] consists of the same 21 items, which measure three factors of emotional reactivity: emotional sensitivity (e.g., “My feelings get hurt easily”), intensity (e.g., “I experience emotions very strongly.”), and persistence (e.g., “When something happens that upsets me, it's all I can think about it for a long time.”). A 5-point Likert scale ranging from 0 “not at all like me” to 4 “completely like me” is used to rate each item. The questionnaire shows good internal consistency of the total score (Cronbach’s α = 0.94 in the original questionnaire and α = 0.94 in the current sample) and its three subscales (Sensitivity: α = 0.88 in the original questionnaire and α = 0.88 in the current sample; Intensity: α = 0.86 in the original questionnaire and α = 0.84 in the current sample; Persistence: α = 0.81 in the original questionnaire and α = 0.76 in the current sample).

For validity testing, participants completed five additional questionnaires. To evaluate convergent validity with general psychological distress, we used the German version of the Symptom-Checklist-K-9 (SCL-K-9, [18–20]. To explore associations between the ERS and the behavioral inhibition/ activation system, we used the Behavioral Inhibition Scale (BIS)/Behavioral Activation Scale (BAS) [21, 22]. The BIS was used for assessing convergent validity, while the three subscales of the BAS were used for assessing discriminant validity. Additionally, we used the Adult Temperament Questionnaire (ATQ; [23, 24] for assessing convergent validity (utilizing the factor scales negative affect and orienting sensitivity) and discriminant validity (utilizing the factor scales effortful control and extraversion/surgency). To examine the convergence between depressive symptoms and symptoms of disordered eating with the ERS, we used the Beck Depression Inventory (BDI-II; [25, 26] and the total score of the Eating Disorder Inventory-II (EDI-II; [27, 28], respectively. Finally, to assess discriminant validity between unhealthy alcohol use and the ERS, we used the total score of the Alcohol Use Disorders Identification Test (AUDIT; [29, 30]. Detailed information on all study questionnaires is available in the supplemental material.

### Data analytic procedures

We used confirmatory factor analysis (CFA) to assess the fit of three different structural models. First, we tested a first-order factor model with three correlated first-order factors. Then, we tested a single-factor model, where all 21 items loaded onto a single overarching factor. Finally, we tested whether the data could be best represented by a bifactor model. This bifactor model consisted of a general factor that loaded directly onto all indicators in the model. In addition, it includes three first-order factors that loaded onto a subset of the same indicators. The first-order factors are orthogonal in the model (see supplemental material for an illustration of the model).

Due to minor deviation from normality of the data and due to the ordinal nature of the items, robust maximum likelihood estimation was used. Model fit was evaluated using chi square statistics (χ2), Comparative Fit Index (CFI), Tucker-Lewis Index (TLI), root mean square error of approximation (RMSEA), and standardized root mean square residual (SRMR). For CFI and TLI, values exceeding 0.95 indicated a good fit, and values above 0.90 suggested an adequate fit. SRMR values around 0.08 or lower indicated a good fit to the data. In the case of RMSEA, values below 0.06 were considered a good fit, while values below 0.08 suggested an adequate fit [31]. Additionally, we used Akaike’s Information Criterion (AIC) and Bayes Information Criterion (BIC) indices to determine the best-fitting model, with the smallest AIC and BIC values indicating the best model fit.

For the model with the best fit, measurement invariance tests were conducted across gender to assess the equivalence of the construct across groups. A sequential strategy was used to test the invariance at different levels. First, in order to establish equivalence in factor structure across the two groups (configural) model, all parameters were freely estimated across groups. Second, a metric model was fitted and compared to the configural model. In the metric model, the factor loadings were constrained to be equal. Third, a scalar model was fitted, in which factor loadings and item intercepts were constrained to be equal, which was compared to the second (metric) model. Fourth, a strict model was fitted, in which factor loadings, intercepts, and residual variances were constrained to be equal. This final model was compared to the third (scalar) model. We report Yuan-Bentler scaled difference chi square test statistic in comparing competing nested models. Even though a scaled chi-square difference test for nested models can be used to index invariance between models, it suffers from the same dependency on sample size as the minimum fit function statistic. Thus, changes in model fit according to CFI, RMSEA and SRMR were used. According to the criteria suggested by Chen [32], a decrease in CFI of ≥ -0.01 in addition to an increase in RMSEA of ≥ 0.015 and SRMR ≥ 0.030 corresponds to an adequate criterion indicating a decrement in fit between models for sample sizes > 300.

The CFAs were carried out using the R (R Core Team, 2018) package “lavaan” [33, 34]. All other statistical analyses were performed using SPSS Version 27.

Spearman correlations were calculated to evaluate relationships between the ERS and all other study questionnaires (SCLK-9, BIS/BAS, BDI-II, EDI-II, AUDIT, ATQ). All tests were conducted two-sided. The strength of the correlations was categorized according to the guidelines by Evans (negligible = 0.00—0.19, weak = 0.20—0.39, moderate = 0.40—0.59, strong = 0.60—0.79, very strong = 0.80- 1.00) [35].

Materials and analysis code for this study are available by emailing the corresponding author.

## Results

### Model fit evaluation

The goodness-of-fit indices for the models of the CFAs are presented in Table 2. The three-factor structure model showed that none of the indices provided an acceptable fit, and hence, no support for the originally proposed three-factor structure model. Similarly, the single-factor model did not show evidence of an acceptable fit. However, the bifactor model showed close to adequate model fit with respect to the CFI, TLI and RMSEA, and good model fit with respect to the SRMR. Thus, the bifactor model was acceptable.

**Table 2:** Estimates of confirmatory factor analyses: model-fit indices for a one-factor model, a three-factor model and bifactor model

Model	χ^2^ (df)	CFA	TLI	RMSEA (CI 90)	SRMR	AIC	BIC
Three-factor	1019.12 (186)	.767	.737	.119 (.112-.126)	.083	20285.25	20459.88
One-factor	1047.39 (189)	.759	.732	.120 (.113-.127)	.083	20314.76	20447.74
Bifactor	**549.85 (168)**	**.894**	**.868**	**.084 (.076-.092)**	**.066**	**19783.69**	**20028.16**

*χ*2 Yuan-Bentler scaled Chi-square (*df* = degrees of freedom), *CFI* Comparative Fit Index, *TLI* Tucker-Lewis index, *RMSEA* Root Mean Square Error of Approximation and 90% confidence interval, *SRMR* Standardized Root Mean Square Residual, *AIC* Akaike’s Information Criterion, *BIC* Bayes Information Criterion. Bold indicates the best fitting factor solution

In the bifactor model, only 5 items continued to robustly load onto their respective domain-specific factors after controlling for the general factor (see Table 3). The factor loadings on the general factor were all lager than 0.40 (0.414—0.750) and, apart from three cases, greater than the loading of the domain-specific factors. Thus, the bifactor model produced the most favorable fit statistics, and the general factor that explains the common variance of the ERS can thus be named “general emotional reactivity”.

**Table 3:** Standardized factor loadings for the bifactor model, and item, factor and model-based reliability indices

Items	G	Int	Sens	Per	I-ECV
Wenn ich Emotionen erlebe, fühle ich diese sehr stark / intensiv	.544	.645			.416
Wenn ich emotional aufgebracht bin, wird mein ganzer Körper ebenfalls physisch aufgeregt	.414	.249			.734
Ich erlebe Emotionen sehr stark	.603	.724			.410
Andere sagen mir, dass meine Emotionen oftmals zu intensiv für die Situation sind	.745	-.129			.971
Meine Stimmungen sind sehr stark und mächtig	.750	.113			.978
Ich rege mich häufig so sehr auf, dass es mir schwer fällt, klar zu denken	.736	-.201			.931
Andere Menschen sagen mir, dass ich überreagiere	.697	-.251			.885
Meine Gefühle werden leicht verletzt	.630		.438		.674
Ich neige dazu, sehr leicht sehr emotional zu werden	.708		.335		.817
Ich fühle mich oft extrem ängstlich	.628		.167		.934
Selbst die kleinsten Dinge lassen mich emotional werden	.688		.204		.919
Ich werde sehr leicht wütend auf andere	.604		-.297		.805
Oftmals beschäftigen mich Dinge, auf die andere Menschen nicht reagieren	.564		.183		.905
Ich gerate leicht in Aufregung	.675		.091		.982
Meine Emotionen wechseln in einem Moment von neutral zu extrem	.705		-.194		.930
Wenn etwas Schlimmes passiert, verändert sich meine Stimmung sehr schnell. Andere sagen mir, dass ich schnell explodiere	.748		-.341		.828
Ich bin eine sehr sensible Person	.528		.442		.588
Wenn etwas passiert, das mich aufbringt, ist es das einzige, worüber ich für lange Zeit nachdenken kann	.527			.257	.808
Wenn ich mich emotional fühle, fällt es mir schwer, mir vorzustellen, mich in irgendeiner Weise anders zu fühlen	.588			.175	.919
Wenn ich mit jemandem eine Meinungsverschiedenheit habe, brauche ich viel Zeit, um darüber hinwegzukommen	.533			.644	.407
Wenn ich wütend/aufgebracht bin, brauche ich viel länger als die meisten Menschen, um mich zu beruhigen	.664			.267	.861
${\varvec{\upomega}}$	.948	.881	.898	.776	-
${\varvec{\upomega}}$_**H**_	.926	.054	.022	.196	-
**ECV**	.769	.102	.077	.052	-

*G* General factor, *Int* Intensity, *Sens* Sensitivity, *Per* Persistence, coefficient omega (*ω*); omega hierarchical (*ωH*), *ECV* Explained common variance, *I-ECV* Item explained common variance

### Evaluation of the Bifactor model

To further evaluate the bifactor model, the *BifactorIndicesCalculator* [36] was used to calculate several additional indices: [1] coefficient omega (ω), [2] omega hierarchical (ωH), (3) explained common variance (ECV), (4) item for explained common variance (I-ECV), and (5) percent uncontaminated correlations (PUC). Subsequently, we provide descriptions of the indices and guidelines regarding evaluation according to Rodriguez and colleagues [37, 38].

*Omega* (*ωS*) is used as a measure of reliability and an analogue to coefficient alpha, as it reflects the proportion of total variance that is attributable to common sources of variance. *Omega hierarchical* (ωH) is used to determine the proportion of reliable variance (i.e., error-free variance) in observed total scores attributable solely to the general factor. These principles can also be applied to specific factors, demonstrating the reliability of a subscale score after controlling for the variance due to the general factor. As evident by the omega estimates presented in Table 3, the values of ω for both the general factor and the three domain-specific factors indicated that they sufficiently explained the common variance among all items. However, the values of ωH indicated that a relatively large proportion of variance was attributed to the general factor.

Explained Common Variance (ECV) and Item for Explained Common Variance (I-ECV) provides a measure of the proportion of variance in test scores that is explained by the general factor compared to the specific factors. This measure ranges from 0 to 1, with values closer to 1 reflecting a ‘stronger’ general factor. When ECV values are > 0.70, the common variance is indicative of unidimensionality. Regarding I-ECV, item loadings on the general factor ≥ 0.80 yield a fairly unidimensional item set that reflects the content of the general dimension. As evident in Table 3, 76.9% of the common variance was attributed to the general factor, while the residual 22.1% of the common variance was attributed to domain-specific factors. As an assessment of unidimensionality at an individual item level (I-ECV), apart from six cases, all other loadings exceeded 0.80, indicating an unidimensional item set that reflects the general dimension.

The Percent of Uncontaminated Correlations (PUC) corresponds to the percentage of covariance terms that exclusively reflect variance from the general dimension. In other words, it captures the extent to which the measurement remains ‘uncontaminated’ by the multidimensionality introduced by the sub-scales. Along with ECV, the PUC influences the parameter bias of the unidimensional solution. As a guideline, “when ECV is > 0.70 and PUC is > 0.70, relative bias will be slight, and the common variance can be regarded as essentially unidimensional [38]”. The PUC value for the ERS was 0.657, indicating that a substantial proportion of the correlations within the ERS is attributable to the general factor. However, it slightly fails to exceed the 0.70 cut-off. Nevertheless, considering the values of the general factor (specifically ωH = 0.926 and ECV = 0.769), it suggests that while some level of multidimensionality is present (according to the PUC) it is not pronounced enough to argue against the unidimensionalitylity of the instrument.

### Invariance testing

Measurement invariance tests were conducted tot test invariance across gender regarding the bifactor model. Analyses (see Table 4 for estimates) showed support for configural invariance (suggesting a similar factor structure across gender). Furthermore, there was no substantial decrease of model fit in both the metric model (indicating the equivalence of the relationship between items and constructs across gender) and the scalar invariance model (indicating that item intercepts are equivalent across gender), indicating that full metric invariance was achieved. Finally, there was support for residual invariance, (i.e., the residuals for the items are equivalent across gender; see Table 4 for estimates).

**Table 4:** Results of the multi-group tests of invariance for gender. Deltas represent change in relation to the previous level of measurement invariance

**Model**	**Δ*χ***^**2**^** (**Δ**df)**	**CFI**	**ΔCFI**	**RMSEA**	**ΔRMSEA**	**SRMR**	**Δ SRMR**
Configural	-	.884	-	.086	-	.071	-
Metric	28.43 (38)	.884	.000	.082	.004	.079	.008
Scalar	20.33 (17)	.884	.000	.080	-.002	.080	.001
Residual	54.37 (21)	.874	.010	.081	.001	.081	.001

Δ*χ*^2^ Yuan–Bentler scaled difference, *CFI* Comparative Fit Index, *TLI* Tucker-Lewis index, RMSEA Root Mean Square Error of Approximation, *SRMR* Standardized Root Mean Square Residual

### Validity testing

There were positive correlations between the total score and all subscales of the ERS as well as between the total ERS score and the BIS, ATQ_OS, ATQ_NA, EDI-II, BDI-II and the SCLK9. Conversely, there were negative correlations between the total score and all subscales of the ERS and the ATQ_EC and the ATQ-EV. For the BAS_R, there were positive correlations with the ERS total score, the sensitivity subscale, and the intensity subscale but not with the persistence subscale. For the BAS_TS, there was only a positive correlation with the persistence subscale. Neither the total ERS score nor the ERS subscales correlated with the AUDIT, the BAS_D or the BAS_F. Correlation coefficients are reported in Table 5.

**Table 5:** Spearman correlations between the ERS and all other questionnaires

		*N*	*M*	*SD*	1	2	3	4	5	6	7	8	9	10	11	12	13	14	15	16	17
1	ERS_T	334	32	16	-																
2	ERS_S	334	15	8	.967^**^	-															
3	ERS_I	334	11	6	.916^**^	.829^**^	-														
4	ERS_P	334	6	4	.826^**^	.738^**^	.654^**^	-													
5	BAS_D	334	12	2.27	-.029	-.044	.016	-.043	-												
6	BAS_F	334	12	2.09	.023	.008	.062	-.030	.179^**^	-											
7	BAS_R	334	16	2.11	.145^**^	.125^*^	.190^**^	.064	.436^**^	.322^**^	-										
8	BAS_TS	334	39	4.75	.071	.050	.125^*^	.006	.730^**^	.651^**^	.789^**^	-									
9	BIS	334	21	3.89	.617^**^	.633^**^	.472^**^	.577^**^	-.075	-.205^**^	.131^*^	-.069	-								
10	ATQ_NA	334	99	22	.722^**^	.721^**^	.609^**^	.635^**^	-.014	-.022	.092	.031	.613^**^	-							
11	ATQ_EC	334	86	15	-.434^**^	-.417^**^	-.379^**^	-.411^**^	.219^**^	-.207^**^	-.035	-.012	-.338^**^	-.475^**^	-						
12	ATQ_EV	334	77	14	-.288^**^	-.293^**^	-.189^**^	-.338^**^	.132^*^	.357^**^	.274^**^	.332^**^	-.251^**^	-.411^**^	.148^**^	-					
13	ATQ_OS	334	69	13	.302^**^	.270^**^	.328^**^	.221^**^	.008	.090	.163^**^	.107^*^	.122^*^	.295^**^	-.133^*^	-.023	-				
14	SCLK9	334	9	6	.553^**^	.549^**^	.493^**^	.464^**^	-.124^*^	-.024	.027	-.045	.446^**^	.559^**^	-.464^**^	-.298^**^	.192^**^	-			
15	BDI-II	334	8	8	.420^**^	.417^**^	.330^**^	.401^**^	-.126^*^	-.056	-.069	-.116^*^	.375^**^	.437^**^	-.442^**^	-.322^**^	.181^**^	.652^**^	-		
16	EDI-II	334	115	23	.348^**^	.313^**^	.318^**^	.326^**^	.173^**^	.100	.212^**^	.216^**^	.255^**^	.358^**^	-.268^**^	-.157^**^	.131^*^	.392^**^	.405^**^	-	
17	AUDIT	334	4	5	.071	.049	.089	.072	-.136^*^	.199^**^	-.006	.014	-.013	.032	-.279^**^	.187^**^	.026	.140^*^	.221^**^	.189^**^	-

*M *Median, *SD *Standard deviation, *ERS_T* Emotion Reactivity Scale, total score, *ERS_S* Emotion Reactivity Scale, sensitivity, *ERS_I* Emotion Reactivity Scale, intensity, *ERS_P* Emotion Reactivity Scale, persistence, *BAS_D* Behavioural Activation Scale, drive, *BAS_F* Behavioural Activation Scale, fun, *BAS_R* Behavioural Activation Scale, reward, *BAS_TS* Behavioural Activation Scale, total score, *BIS* Behavioural Inhibition Scale, *ATQ_NA* Adult Temperament Questionnaire, negative affect, *ATQ_EC* Adult Temperament Questionnaire, effortful control, *ATQ_EV* Adult Temperament Questionnaire, extraversion, *ATQ_OS* Adult Temperament Questionnaire, orienting sensitivity, *SCLK9* Symptom-Checklist-K-9, *BDI-II* Beck Depression Inventory, *EDI-II* Eating Disorder Inventory, *AUDIT* Alcohol Use Disorders Identification Test. * = *p* < .05; ** = *p* < .01

## Discussion

The psychometric evaluation of the German ERS showed the strongest support for a bifactor model of emotional reactivity. In contrast, little support was found for both the three correlated first-order factor model and a single-factor model. Furthermore, the results provided evidence for a unidimensional construct within the bifactor model that was consistent across gender, as indicated by measurement invariance tests. Overall, the results suggest satisfactory construct validity as well as good reliability for the German ERS. In line with previous research [1, 12, 13], there was evidence of convergence between the ERS and other measures, such as the BIS, SCLK9, the ATQ subscales negative affect and orienting sensitivity, BDI-II and EDI-II. Furthermore, there was evidence of discrimination between the ERS and the ATQ subscales extraversion and effortful control, BAS fun and drive and the AUDIT. However, there were mixed results concerning the reward subscale of the BAS.

### Factor structure

For the first time, this study assessed and found support for a bifactor model of the ERS. This is in contrast to previous validation studies, which found support for both a traditional correlated three-factor model and a single-factor model [1, 12–14]. While a traditional correlated first-order factor model only considers that the variance of each item is separately explained by the correlated factors, the bifactor model also specifies the variance in both domain-specific factors and a general factor. Furthermore, the bifactor model determines whether the item response data has a sufficiently strong general factor to justify a unidimensional measurement model [37, 38].

There is no straightforward explanation for the poor model fits of the correlated three-factor or the single-factor model in the present study compared to the other studies using CFA. One reason for poor model fit for the single-factor and the three- factor model could be due be local dependencies among observed variables in the data (some items are relatively highly correlated with each other). A bifactor model accounts for this by allowing for specific factors to capture unique variance in these correlated items. While this study showed that the ERS can be described as a scale consisting of a general factor capturing emotional reactivity and three specific factors capturing unique, but relatively smaller, portions of the variance related to intensity, sensitivity; and persistence, more research regarding the factorial structure is needed. With respect to intensity, 2 items (out of 7 items within the factor) continued to robustly load onto its domain-specific factor after controlling for the general factor. With respect to sensitivity, 3 items (out of 10 items within the factor) moderately loaded onto its domain-specific factor, and 1 item for persistence continued to robustly load onto its domain-specific factor after controlling for the general factor (see Table 3). Thus, while the data suggested strong support for a general factor, there is some evidence of multidimensionality in the scale. However, the previous validation studies [1, 12–14] did not differentiate between a three-factor and a single-factor model when assessing model fit indices. In this context, it is important to note that a correlated factor model may exhibit good overall model fit even in the absence of good local fit. This can be due to strong tendency for cross-loadings in the data, which compromises discriminant validity. Consequently, this can produce model fit estimates similar to the single-factor model. It is difficult to compare this study to previous studies, primarily because several conventional fit measures, including CFI, RMSEA, and SRMR, were not reported in previous studies,. Morevoer, while Lannoy and colleagues [13] did not report the RMSEA, Claes et al. [12] reported none of them. Furthermore, in the study by Claes et al. [12], the reported df for the three-factor model is much smaller than the specification of the model suggests, indicating either a typing error or a misspecification of the model (too many parameters estimated). Another methodological concern is related to the choice of estimation methods. While Claes et al. [12] used robust maximum likelihood estimation to address the ordinal nature of the data, Lannoy et al. [13] used the unweighted least squares (ULS) estimation method. Results from a simulation study by Xia and Yang [39] suggest that when analyzing ordinal data, ULS tends not to adequately detect model misfit.

The unidimensionality of a general factor was preferred based on the analyses of the bifactor solution, which demonstrated superior goodness of fit compared to an alternative unidimensional model, specifically the single-factor model. Consequently, the bifactor model was selected as the best representation of the ERS. However, it is essential to clarify that the superior performance of the bifactor model does not necessarily result from its ability to better capture a broad range of valid response variations. Instead, it seems superior to the alternative model because it accommodates unwanted sources of variability or noise [40]. Nevertheless, given the growing evidence for the general factor of the instrument, one could argue that employing 21 items to assess a single construct may be redundant. Given that a bifactor model allows for a comprehensive assessment of the extent to which each indicator measures shared versus distinct aspects of the construct, applying a bifactor model can inform researchers on the psychometric structure of a measure. This approach can help disentangle the unique variance within each indicator and provide a basis for reducing the numbers of items in the scale.

### Construct validity

Consistent with prior research that has established associations between emotional reactivity and several psychopathological symptoms [1, 6, 41–43], we found moderate convergence between the ERS and the SCL-K-9, which is a brief measure of psychological distress. We also found moderate convergence between the BIS and emotional reactivity, in line with previous findings [1]. Consistent with prior research [1], there were no significant associations between the ERS and the two subscales fun and drive of the BAS, which is indicative of discriminant validity of the ERS. However, our findings differ from those of previous research concerning the subscale reward responsiveness and the total score of the BAS.

In line with findings of two previous studies [1, 12], we had hypothesized that the ERS would show convergence with the subscales negative affect and orienting sensitivity of the ATQ and divergence with the two subscales effortful control and extraversion of the ATQ. However, while we found moderate convergence between the ERS and the subscale negative affect, the association between the ERS and the subscale orienting sensitivity was weak. Furthermore, there were weak negative correlations between the ERS and the subscales effortful control and extraversion, which differs from what previous studies reported [1, 12]. Considering that different facets of temperament also correlate with personality traits [24], it would be of importance for future studies to explore the extent to which emotional reactivity correlates with different characteristics of personality.

Several studies have reported positive correlations between emotional reactivity and affective disorders [1, 12, 14, 41, 44], which is why we expected that the BDI-II would show convergence with the German ERS, as confirmed by the present results. It would be of importance to further investigate whether emotional reactivity might predispose individuals to develop depressive symptoms or whether depressive symptoms might intensify emotional reactivity, leading individuals with such symptoms to experience emotions more quickly, intensely and for a longer time. We furthermore successfully replicated convergence between symptoms of eating disorders (using the EDI-II) and the ERS [12, 45]. However, it is worth noting that this association was relatively weak in the present study.

We used the AUDIT to test for discriminant validity with the ERS, since no association of alcohol consumption and the ERS had been found so far [1]. Although we replicated the findings of Nock and colleagues [1], it should be noted that some studies have suggested a potential association between emotional reactivity and alcohol consumption [12, 46–48]. Consequently, more research is needed to investigate whether there is a robust association between alcohol consumption and emotional reactivity.

### Limitations and constraints on generality

The present study is not without limitations. The questionnaire was applied using an online tool, limiting the ability to control for low data quality. However, results from all questionnaires were inspected manually to decrease such potential bias. Concerning the study sample, most individuals were young adults with a high level of educational. Although there were only weak correlations between ERS scores, age and educational level, the generalizability of our findings across educational background, age groups, and, notably, ethnicity (which was not assessed) is limited. Moreover, prevalence of diagnosed mental disorders in our study population was low and we did not distinguish between individuals from the general population and those from clinical settings. Therefore, future studies should aim to investigate emotional reactivity in clinical samples. Additionally, the questionnaire was translated by bilingual individuals who were familiar with the topic of research. Lastly, results relied solely on self-report measures, with no objective assessments like heart rate or blood pressure changes [49]. Future studies should aim to implement both subjective and objective measures to comprehensively assess emotional reactivity.

## Conclusion

Overall, this study provides strong support that the ERS should be treated as a unidimensional construct and confirms the reliability and validity of the German version of the ERS. Thus, the questionnaire can be used in clinical and research settings. Higher levels of emotional reactivity have been consistently associated with a range of mental health problems (e.g., symptoms of eating disorders and affective disorders [41, 44, 45] which merits further investigation. Based on findings of this study, future research should consider the development a short version of the ERS.

### Supplementary Information

**Additional file 1:** **Appendix A.** Additional Questionnaire Information. **Appendix B. Figure 1. **Illustration of three alternative factor structure models for the ERS. **Appendix C.** German Emotion Reactivity Scale.

## Data Availability

The datasets used and/or analyzed during the current study are available from the corresponding author on reasonable request.

## Abbreviations

ERS: Emotional Reactivity Scale
EI: Emotion Intensity
ES: Emotion Sensitivity
EP: Emotion Persistence
SCL-K-9: Symptom-Checklist-K-9
BIS: Behavioral Inhibition Scale
BAS: Behavioral Activation Scale
ATQ: Adult Temperament Questionnaire
BDI-II: Beck Depression Inventory
EDI-II: Eating Disorder Inventory-II
AUDIT: Alcohol Use Disorders Identification Test
CFA: Confirmatory Factor Analysis (CFA)
CFI: Comparative Fit Index
TLI: Tucker-Lewis Index
RMSEA: Root Mean Square Error of Approximation
SRMR: Standardized Root Mean Square Residual
AIC: Akaike’s Information Criterion
BIC: Bayes Information Criterion
ECV: Explained Common Variance
I-ECV: Item for Explained Common Variance
PUC: Percent Uncontaminated Correlations
ULS: Unweighted Least Squares
