# Supplementary material for: Construct validity of the German version of the Emotion Reactivity Scale
Source: BMC Psychol. 2023 Dec 2;11:423. doi: 10.1186/s40359-023-01458-y (PMC10693029; doi:10.1186/s40359-023-01458-y)
Supplement: Supplementary file 1 — Additional file 1: Appendix A. Additional Questionnaire Information. Appendix B. Figure 1. Illustration of three alternative factor structure models for the ERS. Appendix C. German Emotion Reactivity Scale. [file 40359_2023_1458_MOESM1_ESM.docx]

**Appendix A**

**Additional Questionnaire Information**

***Symptom-Checklist-K-9*.** The Symptom-Checklist-K-9 (SCL-K-9, (1,2) is a short form of the SCL-90-R. It is an efficient, unidimensional screening tool to assess psychological distress using 9 items (1,2). All questions are rated on a 4-point scale ranging from 0 “not at all” to 4 “extremely” leaving each participant with a total score between 0 and 36 points. The questionnaire has been validated within a representative German sample (N = 2,057), showing good internal consistency (Cronbach α = 0.87 in the original questionnaire, α =.83 in the current sample), and validity with other measures of psychopathology (e.g., depression) (1,2). In this study, we used the German version developed by Prinz and colleagues (3). The total score obtained within the SCL-K-9 was used to measure convergent validity.

***Behavioral Inhibition Scale/Behavioral Activation Scale*.** The Behavioral Inhibition Scale (BIS)/Behavioral Activation Scale (BAS) is self-report questionnaire of 24 items (4). It measures both the behavioral inhibition system (BIS; motivation to avoid aversive outcomes) and the behavioral activation system (BAS; motivation to approach goal-oriented outcomes). For this study, we used the German version developed by Strobel and colleagues (5) which showed acceptable internal consistency (Cronbachs α for BIS = .78 and for the BAS α = .81 in the original questionnaire, in the current sample Cronbachs α for the BIS α = .76 and α = .72 for BAS total score). Each item is rated on a 4-point Likert scale: 1 (very false for me) 2 (somewhat false for me), 3 (somewhat true for me), and 4 (very true for me). The BIS/BAS consists of four subscales, wherein one subscale corresponds to the BIS and three subscales correspond to three separate components of BAS: 1) Drive (motivation to follow one’s goals) 2) Reward Responsiveness (sensitivity to pleasant reinforcers in the environment) and 3) Fun Seeking (motivation to find novel rewards spontaneously). Elevated scores within the BIS reflect a higher awareness of negative events. The BIS was used to determine convergent validity while the total score and the three subscales of the BAS were used to investigate discriminant validity with the ERS.

***Adult-Temperament-Questionnaire-Short-Form*.** The Adult Temperament Questionnaire (ATQ; (6) serves as a tool to assess four so-called factor scales with each of them consisting of three or four subscales: 1) effortful control (attentional control, inhibitory control and activation control) 2) negative affect (fear, sadness, discomfort and frustration), 3) extraversion/surgency (sociability, positive affect and high intensity pleasure), and 4) orienting sensitivity (neutral perceptual sensitivity, affective perceptual sensitivity and associative sensitivity). In this study, we used the German version developed by Wiltink and colleagues (7) which showed good internal consistency (Cronbach’s α between .72 and .84 in the original questionnaire, in the current sample Cronbach’s α between .65 and .86). All questions are rated on a scale ranging from 1 “not at all applicable” to 7 “completely applicable”. We used the scales negative affect and orienting sensitivity to assess convergent validity and the factor scales effortful control and extraversion/surgency to assess discriminant validity.

***Beck Depression Inventory****.* The Beck Depression Inventory (BDI-II; (8) is a 21-item self-report questionnaire measuring the severity of depression. All items consist of four statements with increasing intensity (e.g., 0 “I do not feel sad”, 1 “I feel sad”, 2 “I am sad all the time and I can't snap out of it”, 3 “I am so sad and unhappy that I can't stand it”). All questions refer to the last two weeks. For this study, we used the German version of the BDI-II developed by Kühner and colleagues which showed good internal consistency (Cronbach’s alpha ≥ 0.84, in the current sample Cronbach’s α = .93) (9). We used the BDI-II to assess convergent validity with the ERS.

***Eating Disorder Inventory II*.** The Eating Disorder Inventory-II (EDI-II; (10,11) is a self-report instrument for assessing disordered eating behavior and associated psychological characteristics. The questionnaire consists of 64 items divided into eight subscales: drive for thinness, bulimia, body dissatisfaction, ineffectiveness, perfectionism, interpersonal distrust, interoception and maturity fears. For this study, we used the German version developed by Thiel and colleagues (11) which showed sufficient internal consistency (Cronbachs α ranging from 0.58 to 0.90 in the original questionnaire, in the current sample Cronbachs α = .95). In the German version, three new subscales have been introduced which we did not incorporate into this survey due to only partially confirmed reliability and validity. We only used the eight original subscales as described above. Each question is rated on a scale ranging from 1 “never” to 6 “always”. Based on previous research (11) we used the total score of the EDI-II to assess convergent validity of the ERS.

***Alcohol Use Disorders Identification Test*.** The Alcohol Use Disorders Identification Test (AUDIT; (12,13) is a screening tool for unhealthy alcohol use (risky or hazardous consumption and any alcohol use disorder) consisting of 10 questions. In this study, we used the German version developed by Dybek and colleagues (13) which showed good reliability (intraclass correlation coefficient was .95 for the total score, Cronbachs α in the current sample = .84). Each question is rated on a scale ranging from 0 “never” to 4 “4 or more times a week”. We used the total score to test for discriminant validity of the ERS.

**Appendix B**

**Figure 1**

Illustration of three alternative factor structure models for the ERS

**
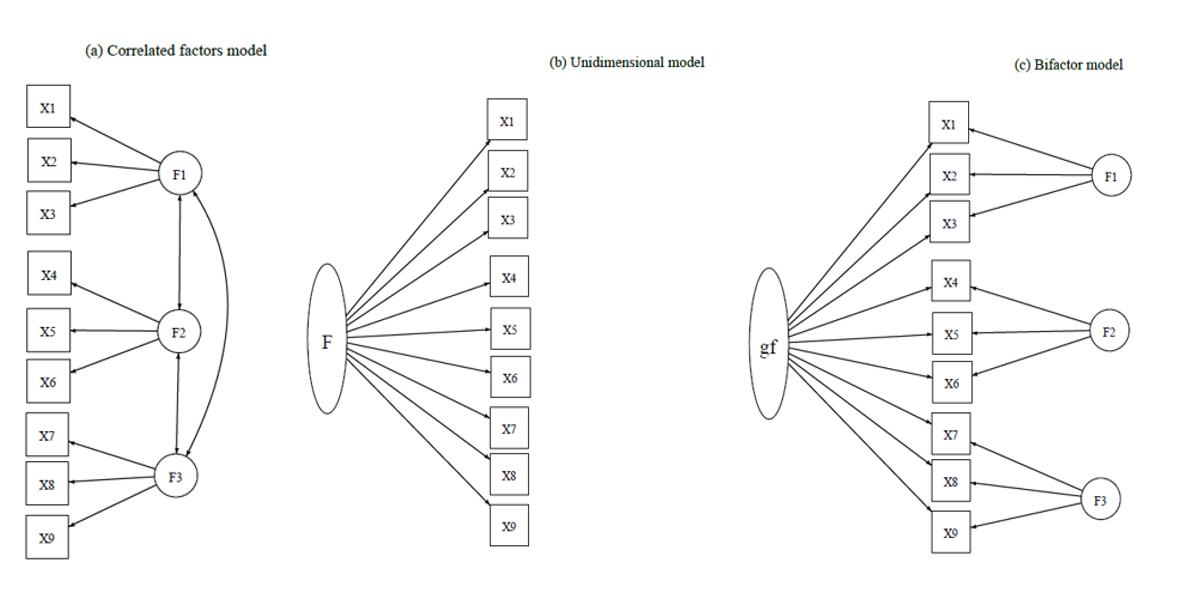
**

*Note.* Illustration (using only 9 indicators for illustrative purposes) of three alternative factor structure models for the ERS (from left to right): the correlated three-factor model (a) the unidimensional model (b), the bifactor model (c).

**Appendix C**

**German Emotion Reactivity Scale**

Dieser Fragebogen stellt unterschiedliche Fragen dazu, wie Sie Emotionen im Allgemeinen erleben. Wenn Sie gefragt werden, ob sie „emotional“ sind, kann sich dies darauf beziehen, dass Sie wütend, traurig oder aufgeregt sind oder eine andere Emotion empfinden. Bitte bewerten Sie die folgenden Aussagen.

|  | 0 = überhaupt nicht typisch für mich | 1 | 2 | 3 | 4 = absolut typisch für mich |
| --- | --- | --- | --- | --- | --- |
| Wenn etwas passiert, das mich aufbringt, ist es das einzige, worüber ich für lange Zeit nachdenken kann. |  |  |  |  |  |
| Meine Gefühle werden leicht verletzt. |  |  |  |  |  |
| Wenn ich Emotionen erlebe, fühle ich diese sehr stark / intensiv. |  |  |  |  |  |
| Wenn ich emotional aufgebracht bin, wird mein ganzer Körper ebenfalls physisch aufgeregt. |  |  |  |  |  |
| Ich neige dazu, sehr leicht sehr emotional zu werden. |  |  |  |  |  |
| Ich erlebe Emotionen sehr stark. |  |  |  |  |  |
| Ich fühle mich oft extrem ängstlich. |  |  |  |  |  |
| Wenn ich mich emotional fühle, fällt es mir schwer, mir vorzustellen, mich in irgendeiner Weise anders zu fühlen. |  |  |  |  |  |
| Selbst die kleinsten Dinge lassen mich emotional werden. |  |  |  |  |  |
| Wenn ich mit jemandem eine Meinungsverschiedenheit habe, brauche ich viel Zeit, um darüber hinwegzukommen. |  |  |  |  |  |
| Wenn ich wütend/aufgebracht bin, brauche ich viel länger als die meisten Menschen, um mich zu beruhigen. |  |  |  |  |  |
| Ich werde sehr leicht wütend auf andere. |  |  |  |  |  |
| Oftmals beschäftigen mich Dinge, auf die andere Menschen nicht reagieren. |  |  |  |  |  |
| Ich gerate leicht in Aufregung. |  |  |  |  |  |
| Meine Emotionen wechseln in einem Moment von neutral zu extrem. |  |  |  |  |  |
| Wenn etwas Schlimmes passiert, verändert sich meine Stimmung sehr schnell. Andere sagen mir, dass ich schnell explodiere. |  |  |  |  |  |
| Andere sagen mir, dass meine Emotionen oftmals zu intensiv für die Situation sind. |  |  |  |  |  |
| Ich bin eine sehr sensible Person. |  |  |  |  |  |
| Meine Stimmungen sind sehr stark und mächtig. |  |  |  |  |  |
| Ich rege mich häufig so sehr auf, dass es mir schwer fällt, klar zu denken. |  |  |  |  |  |
| Andere Menschen sagen mir, dass ich überreagiere. |  |  |  |  |  |

**References**

1. Klaghofer R, Brähler E. Konstruktion und Teststatistische Prüfung einer Kurzform der SCL-90–R. [Construction and test statistical evaluation of a short version of the SCL-90–R.]. Z Für Klin Psychol Psychiatr Psychother. 2001;49(2):115–24.

2. Petrowski K, Schmalbach B, Kliem S, Hinz A, Brähler E. Symptom-Checklist-K-9: Norm values and factorial structure in a representative German sample. PLoS ONE. 2019;14(4).

3. Prinz U, Nutzinger DO, Schulz H, Petermann F, Braukhaus C, Andreas S. Die Symptom-Checkliste-90-R und ihre Kurzversionen: Psychometrische Analysen bei Patienten mit psychischen Erkrankungen. Phys Med Rehabil Kurortmed. Dezember 2008;18(6):337–43.

4. Carver CS, White TL. Behavioral inhibition, behavioral activation, and affective responses to impending reward and punishment: The BIS/BAS Scales. J Pers Soc Psychol. 1994;67(2):319.

5. Strobel A, Beauducel A, Debener S, Brocke B. Eine deutschsprachige Version des BIS/BAS-Fragebogens von Carver und White. [A German version of Carver and White’s BIS/BAS scales.]. Z Für Differ Diagn Psychol. 2001;22(3):216–27.

6. Evans DE, Rothbart MK. Developing a model for adult temperament. J Res Personal. August 2007;41(4):868–88.

7. Wiltink J, Vogelsang U, Beutel ME. Temperament and personality: the German version of the Adult Temperament Questionnaire (ATQ). GMS Psycho-Soc Med. 11. Dezember 2006;3:Doc10.

8. Beck AT, Steer RA, Brown G. Manual for the Beck Depression Inventory–II [Internet]. San Antonio, Tex, Psychological Corporation; 1996 [zitiert 14. Mai 2021]. Verfügbar unter: https://doi.org/10.1037/t00742-000

9. Kühner C, Bürger C, Keller F, Hautzinger M. Reliabilität und Validität des revidierten Beck-Depressions-inventars (BDI-II). Befunde aus deutschsprachigen Stichproben. [Reliability and validity of the Revised Beck Depression Inventory (BDI-II). Results from German samples.]. Nervenarzt. 2007;78(6):651–6.

10. Garner DM. Eating Disorder Inventory-2: Professional manual. 1991;Psychological Assessment Resources, Inc.

11. Thiel A, Jacobi C, Horstmann S, Paul T, Nutzinger DO, Schüßler G. Eine deutschsprachige Version des Eating Disorder Inventory EDI-2. [German translation of the Eating Disorder Inventory EDI-2.]. PPmP Psychother Psychosom Med Psychol. 1997;47(9–10):365–76.

12. Babor TF, Higgins-Biddle, J. C., Saunders, J. B., Monteiro, M. G. The Alcohol Use Disorders Identification Test: Guidelines for Use in Primary Care (2. Aufl.). 2001;World Health Organization, Department of Mental Health and Substance Abuse Dependence.

13. Dybek I, Bischof G, Grothues J, Reinhardt S, Meyer C, Hapke U, u. a. The reliability and validity of the Alcohol Use Disorders Identification Test (AUDIT) in a German general practice population sample. J Stud Alcohol. Mai 2006;67(3):473–81.
